# Play First Before Doing Your Exercise: Does Acting in a Game-Like Task Improve 5-Year-Olds’ Working Memory Performance?

**DOI:** 10.3389/fpsyg.2021.659020

**Published:** 2021-04-28

**Authors:** Christophe Fitamen, Valérie Camos

**Affiliations:** ^1^Département de Psychologie, Université de Fribourg, Fribourg, Switzerland; ^2^Laboratoire de Psychologie Cognitive, UMR 7290, Université d’Aix-Marseille and CNRS, Marseille, France

**Keywords:** working memory, preschoolers, action, game, goal cue

## Abstract

It has been shown that acting in a game-like task improves preschoolers’ working memory when tested in a reconstruction task. The game context and the motor activity during the game would provide goal cues bringing support to the memory processes. The aim of the present study was to test this hypothesis by examining preschoolers’ working memory performance in a game-like task compared to an exercise-like task, which offers less goal cues. In the present study, 5-year-olds had to maintain a series of fruits and vegetables while acting in a game-like task or remaining static during the same task presented in a school-exercise context (within-subject factor). Memory performance was tested either through oral recall or reconstruction of the series of memory items (between-subject factor). Despite the fact that memory performance did not differ between the two conditions (game vs. exercise) whatever the type of memory tests, performance was worst in the game-like than in the exercise condition when the exercise was presented first. No difference emerged between conditions when the game condition was performed first. This result suggests that preschoolers were able to take advantage of acting in the game-like condition to integrate some task requirements, which were beneficial for performing the exercise condition.

## Introduction

Do your exercise first and then you can go playing! Any child has already heard this. We will not question here the fact of being rewarded for the effort during homework. On the contrary, we will see that starting with a fun activity, like a game, would allow children to benefit from it for the achievement of a following, more academic, activity. The hypothesis we tested in the present study is related to the maintenance of the goal during an activity. In previous studies, it has been shown that preschoolers’ performance in inhibition and cognitive flexibility tasks is impaired when they cannot actively maintain the goal during the task (Marcovitch et al., 2007, 2010; Chevalier and Blaye, 2008; Yanaoka and Saito, 2017). However, it is possible to help preschoolers to effectively maintain the goal during the task by presenting them with meaningful contextual cues related to the goal to be pursued (Towse et al., 2007; Chevalier and Blaye, 2009; Blaye and Chevalier, 2011). Alternatively, it has been shown that some motor activity like gestures provide support to cognitive performance. In the present study, we hypothesized that acting in a game-like context can provide meaningful goal cues because it joins the motor activity and contextual cues that would have a favorable impact on preschoolers’ working memory performance. Before examining the role of goal cues in executive functions, we present the impact of motor activity in supporting working memory.

### Working Memory and Influence of Motor Activity

According to some theoretical conceptions, working memory is part of executive control (Diamond, 2016), and in charge of the storing and processing of information at short term (Baddeley, 1986). Working memory is involved in learning processes such as reading (Cain et al., 2004; Cain, 2006), in text comprehension (Carretti et al., 2005), in arithmetical activities where it predicts subsequent success (De Smedt et al., 2009), in reasoning, and in all other high-level cognitive activities (Camos and Barrouillet, 2018, for a review). As a consequence, working memory capacity is an excellent predictor of academic success (Gathercole and Alloway, 2004), and measures of working memory capacity also provides a better prediction than does the assessment of IQ based, in part, on the assessment of general knowledge as reading skills and mathematics (Alloway, 2009; Alloway and Alloway, 2010). In addition, working memory capacity is a better predictor than socio-economic level (Barrouillet et al., 2008) and does not depend on knowledge acquired before school period (Alloway et al., 2004).

Recently, research in cognitive psychology has investigated the support that motor activity can provide to working memory, especially the role of gestures. For example, in solving additions, children with poorer working memory capacity use strategies such as counting on their fingers to compensate their difficulties (Geary et al., 2004). In the same vein, according to Cook et al. (2012), producing meaningful gestures could reduce the cognitive cost in young adults when they solve a mathematical problem. The help of gestures when solving a math problem has also been observed in children aged 9 and 10 years. More specifically, children exhibited better performance in solving a problem when they received task instructions to use gestures or when the experimenter herself used gestures compared to children who had neither seen gestures from the experimenter nor did gestures (Cook and Goldin-Meadow, 2006). In a study testing 10-year-old children and young adults, Goldin-Meadow et al. (2001) tested that the use of gestures reduced working memory load. Before solving math problems, children and adults were presented with a list of words or letters to be recalled after the problem solving. The authors found that when children and adults were able to use gestures during the problem solving, their recall performance of the memory list was better than when they did not use gestures. Hence, performing gestures during the concurrent task of problem solving would free up cognitive resources for the memory task (see also Goldin-Meadow, 2011). In the same vein, a study by So et al. (2012) involving children aged 4 and 5 years provides evidence in favor of the usefulness of gestures in a verbal memory task. Children were involved in three different conditions of a verbal memory task. Children watched a videotaped narrator who recited a list of verbs and produced meaningful gestures who were iconic gestures, or children saw the narrator reciting the verbs and produced beat gestures simultaneously, or they watched the narrator reciting the verbs without any gesture. When children recalled the verbs after a 2 min delay, their performance was better in the first condition, the two others did not differ. Hence, gestures can help children in improving their performance either on a memory task or on a secondary memory task by freeing cognitive resources during the primary task.

Moreover, it has been shown that another type of motor activity, i.e., walking, can also help memory performance. In a study involving 9-year-old children and young adults, participants were asked to perform an auditory *n*-back (one-back to four-back) task. In this task, participants heard one letter at a time and, for example, in the case of a two-back, they must spot when a new letter is identical to the one presented two letters before. While participants were doing the *n*-back task, they either walked on a treadmill or remained seated (Schaefer et al., 2010). When walking on the treadmill, participants performed either at their preferred pace or at a slower pace than their usual walking speed. Both children and adults exhibited better performance in the *n*-back task when they freely choose their walking speed compared to the slower-pace or remaining-seated conditions. In addition to studies on the role of gestures, this study converges in showing that motor activities can provide support to memory performance in adults, school-age children, and even preschoolers.

In the present study, we would like to suggest that the benefit from the physical involvement of children in performing an activity results from the active maintenance of the task goal, because in many cases, this physical involvement is oriented toward the goal of the task.

### Goal Maintenance by Enactment

Two studies previously conducted by Istomina (1975) and Bertrand and Camos (2015) highlight the importance of the link between performing an action that makes sense in relation to the pursued goal and the active maintenance of that goal. In an old experiment first published in Russian in 1948, Istomina (1975, for the English translation) tested long-term memory in children aged 3–7 years old under two experimental conditions. Children were engaged either in a condition where they remained static and which was akin to a school-exercise condition, or in a condition requiring a motor activity in which they had to take part in a role play, doing shopping. In the exercise condition, children had to listen attentively to the words said by the experimenter to orally recall them after a 60–90 s delay. In the shopping condition, lists of items were presented as a shopping list and children had to go to a toy shop and asked for the items to another child playing the merchant. Preschoolers showed better recall performance in the play than in the exercise condition. The author suggested that the shopping game context emphasized the task goal (memorizing for further recall) through the provision of a pragmatic relevance to recall. Hence, the overall context of the task can provide some cueing that would help to maintain the task goal, resulting in improved memory performance. It is noteworthy that because walking is itself goal-directed, it could also play the role of a goal cue that would support goal maintenance in children. This last interpretation was raised by Bertrand and Camos (2015) in their study, in which the authors implemented a rather similar game situation to assess preschoolers’ working memory. The task was also a shopping game in which 4- to 6-year-old children had to memorize lists of verbal items for further recall either immediately after the presentation of the items or after a delay. Preschoolers’ working memory performance improved when they had to walk straight to a toy shop instead of waiting seated in the front of the shop during the same delay. Among their interpretations, the authors suggested that walking improved goal maintenance in preschoolers and led to the observed better recall performance, because it is a goal-oriented motor activity. To summarize, findings of these two studies suggest that when children’s memory capacity is assessed in a goal-supportive context this can help improving preschoolers’ memory. Moreover, where there is an enactment of a situation, this can provide some support to the goal maintenance leading to improved memory performance.

A recent study by Fitamen et al. (2019) brought further evidence to support this last hypothesis. In a computerized working memory task, 5-year-old children had to memorize lists of items while they watched an animation of either a schoolbag that symbolized the container of the memory items, or a non-meaningful rectangle. Moreover, in two further conditions, children had to follow with their finger the movement on screen of the schoolbag or the rectangle. Children exhibited their best recall scores when they had to track the schoolbag. Hence, the concomitance of contextual cues (the schoolbag) and the motor involvement (tracking) led to improvement in working memory performance. The present study aimed at testing the joint effect of contextual cues and action in improving working memory performance in preschoolers by extending this previous finding into a more natural setting, akin to the situations used in Istomina (1975) and Bertrand and Camos (2015).

### The Present Study

In the present study, we enrolled 5-year-old children in two experimental conditions, one enriched in contextual cues and proposing an oriented motor activity toward the goal (game condition), the other presenting neither contextual cues nor motor activity (exercise condition). In the game condition, those children were involved in a role play of shopping where after memorizing a list of items they walked to a market stall. This condition was similar to Bertrand and Camos (2015). In the exercise condition, children were involved in an exercise situation more comparable to their everyday classroom exercises where they also had to memorize a list of words while sitting in front of experimenters. This condition was comparable to exercise condition of Istomina (1975). We hypothesized that the joint effect of action and contextual cues on goal maintenance in these natural settings should improve memory performance and children should exhibit better recall scores in the game than in the exercise condition.

However, such a beneficial effect of the action and contextual cues on memory performance should occur only when the task does not in itself favor the goal maintenance. In Bertrand and Camos (2015) study as well as in some conditions of Istomina (1975), children performed a reconstruction test. Hence, during the testing phase, children were asked to collect the previously encoded fruits and vegetables in a box containing different elements. This type of tests could encourage goal neglect because children know since the beginning of the task that the items will be presented to them during the testing phase. They may not try to actively maintaining memoranda and the goal during the delay of retention. Moreover, it can be assumed that a reconstruction test can be carried out by appealing only to familiarity of memory traces stored in long-term memory (see Yonelinas, 2002, for a review; Malmberg, 2008) that is without having to actively maintain memory traces in working memory. Thus, the type of tests implemented at the end of a working memory task could impact the goal maintenance. For example, in a Stroop task (Kane and Engle, 2003) and in a card sorting task (Marcovitch et al., 2007), when the task required frequent reactivation of the goal (i.e., predominantly incongruent condition in the former and predominantly conflicting condition in the latter), errors decreased compared to conditions that did not require active goal maintenance (predominantly congruent condition in the former and redundant condition in the latter). Hence, the characteristics of the test can more or less call for goal maintenance. In a reconstruction test that in itself provides retrieval cues at test, goal can be more easily neglected than in an oral recall test in which children had to rely on active maintenance to produce the memory items.

To test this additional hypothesis, we manipulated the type of tests by proposing to 5-year-old children either a reconstruction test or an oral recall test. We expected better memory performance in the reconstruction than in the oral recall test, replicating the difference reported between recall and recognition tests (see Tiberghien and Lecocq, 1983, for a review), although the reconstruction test is situated between recognition and recall as it has to preserve the serial order unlike recognition test, but like recall test. Moreover, we hypothesized that goal neglect would occur in the task with the reconstruction test and not with the oral recall test. Under the reconstruction test, children should then perform better in a play condition that helps goal maintenance that in an exercise condition that did not provide any goal support. However, under the oral recall test, we should not observe any effect of the type of contexts (exercise or game) on children’s working memory performance. Thus, we expected to observe an interaction between the type of tests (reconstruction vs. oral recall) and the type of contexts (exercise vs. game).

## Materials and Methods

### Participants

Sixty-two 5-year-olds (*M*_age_ = 4;11, *SD* = 0;4, 30 girls) took part in the experience. The mother tongue was French for all children. The experiment took place at the children’s school in a quiet location. The experiment was approved by the local ethics committee, and we gathered from the parents or legal guardians a consent form. Children gave also their consent orally before beginning the experiment.

Three children were excluded from the analyses. One was followed in occupational therapy, another in speech therapy, and a last one could not sufficiently maintain his attention during the second experimental condition making the task unworkable. This led to a final sample of 59 children, randomly assigned to the two tests (29 in reconstruction and 30 in recall test).

### Material and Procedure

The design was adapted from Istomina (1975) and Bertrand and Camos (2015). The experiment had a mixed design with the type of tests (reconstruction vs. oral recall) as between-subject factor, and the type of contexts (exercise vs. game) as within-subject factor. The order of presentation of the two conditions of context was counterbalanced.

To assess the similarity of the two groups in working memory capacity, every child performed before the experimental conditions the Number Recall subtest of the K-ABC 2 with 3 series in each length ranging from 2 to 9 digits, except for length 8 with only one series (Kaufman and Kaufman, 1993). Testing stopped after three successive series not correctly recalled. In this subtest, each correctly recalled series gave 1 point, and the raw score was the sum of the point (maximum score = 22). Before the experimental session, we also assessed the distance each child can walk at her own pace in 4 s in one training trial and three test trials. The average distance walked on the test trials determined the walking distance in the game context (see below). The distance was hence adapted to each child (mean = 4 m and *SD* = 1 m).

Nine different experimenters were involved in the study, but only two intervened with each child. One experimenter was in charge of the encoding part while another experimenter took care of the recall part of the working memory task. Before starting the working memory task, the experimenter verified at the encoding that the child recognized each plastic item representing fruits and vegetables. The fruits and vegetables (banana, tomato, orange, lemon, and carrot) were selected to have French bi-syllabic names with high frequency (Lété et al., 2004), an early age of acquisition (in years, 1.58, 1.65, 1.62, 1.88, and 1.58, respectively; Alario and Ferrand, 1999), but also different shapes and colors to be easily distinguished from each other. Children had to memorize lists of 1–4 fruits and vegetables. Four series were presented in each length, a given item appearing only once in each series. However, each item was presented in several series, which prevents that recall relies only memory traces from long-term memory. Two lists of memory series were created, one per condition of context (exercise vs. game) for each child. A trial started when the experimenter took one fruit or vegetable, named it and put it in a transparent tube-shaped bag narrow enough to keep items on top of each other, arranged in a single column, the child paying attention to the scene. The items were successively introduced in the bag at a roughly regular rate of one every second. When all the items of the series were in the experimenter’s bag, the bag was hidden to the child’s eyes. Then, after a 4-s delay, the child had to reproduce the series according to the conditions she was assigned to (see below for the description of the four different experimental conditions). The child proceeded to the next length if she produced perfect recall (i.e., correct fruits and vegetables in correct order) on at least one trial of a given length. Each child had to reproduce series of items in two different conditions (exercise vs. game). Children performed the two conditions in the same room.

For the *exercise condition with oral recall test*, the child stayed seated in front of two experimenters (one for encoding, one for recall) after the “encoding” experimenter’s bag was hidden, and waited for an auditory signal heard after 4-s delay. At the signal, the “recall” experimenter opened an opaque box, placed between the child and the experimenters, and which contained the five different fruits and vegetables that were not visible to the child. Once the child has orally recalled an item, the “recall” experimenter took it from the box and put it in a transparent tube-shaped bag similar to the bag used for the encoding. The *exercise condition with reconstruction test* was similar, except that the box was opened in front of the child so that she could see and grab easily one by one the fruits and vegetables to reconstruct the memorized sequence. The child put herself the fruits and vegetables in her transparent tube-shaped bag during the reconstruction test.

In the two *game conditions* (*reconstruction* and *oral recall*), the child had to walk with their empty bag straight to the shopping stall after the presentation of the items and the signal of the experimenter in charge of encoding to “go ahead.” After 4 s, the recall experimenter who played the merchant opened the box placed on the stall. The child performed the test depending on the condition (*reconstruction* or *oral recall*) in the same way as in the exercise conditions.

A span score was computed for each child in each condition. Each correctly recalled series (i.e., in which all the items were correctly placed in the order of presentation) counted as one-fourth, and the total number of fourths added (Smyth and Scholey, 1992; Barrouillet et al., 2009; Bertrand and Camos, 2015).

## Results

A first ANOVA was performed on the raw scores of the Number Recall subtest of the K-ABC 2 with the type of tests, the lists, and the order of presentation of the type of contexts as between-subject factors. All effects were non-significant, *ps* > 0.10. Importantly for the purpose of the present study, the two groups of children that were randomly assigned to each condition of tests (reconstruction: mean = 7.0, SD = 2.2; oral recall: mean = 6.8, SD = 2.0) did not differ on the Number Recall task, *F*(1,51) = 0.065, *p* = 0.80, *n*_p_^2^ = 0.001.

A second ANOVA was performed on span scores with the type of contexts as within-subject factor, and the type of tests, the lists, the order of presentation of the context conditions as between-subject factors. The only significant effect was the interaction between the type of contexts and its order of presentation, *F*(1,51) = 8.46, *p* = 0.005, *n*_p_^2^ = 0.142. The values of *p* for the other effects were higher than 0.20. It should be noted that the interaction of interest between the type of tests and the type of contexts was non-significant, *F*(1,51) = 0.456, *p* = 0.503, *n*_p_^2^ = 0.009. To take into account individual differences, we added in a third ANOVA the score at the digit span task as covariable. The same pattern of findings emerged as in the previous analysis. As expected, the score at the digit span task had a significant effect on the recall performance of our main tasks, *F*(1,50) = 25.70, *p* < 0.001, *n*_p_^2^ = 0.340. Except this last effect, the only other significant effect was the interaction between the type of contexts and its order of presentation, *F*(1,50) = 8.80, *p* = 0.005, *n*_p_^2^ = 0.150. The interaction of interest between the type of tests and the type of contexts remained non-significant, *F*(1,50) = 0.502, *p* = 0.482, *n*_p_^2^ = 0.010. This absence of interaction was confirmed by the analyses comparing the type of contexts within each type of tests, *t*(50) = 1.43, *p* = 0.16 and *t*(50) = 0.41, *p* = 0.68 in recall and reconstruction tests, respectively.

**Figure 1:**
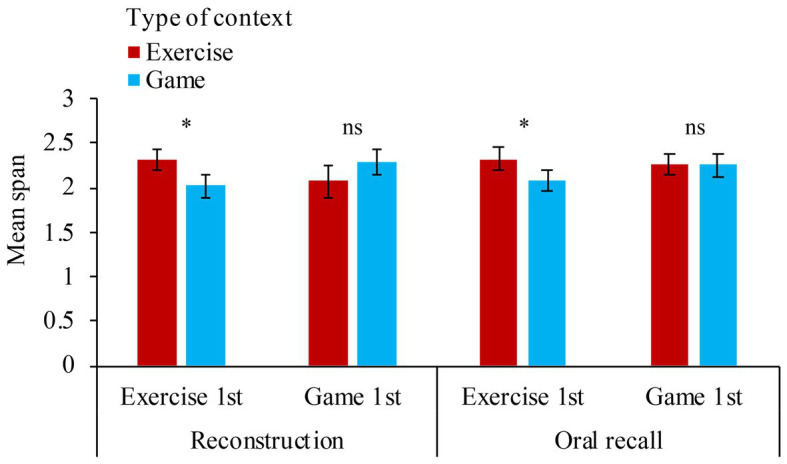
Mean span as a function of the type of contexts (exercise vs. game), the order of presentation of the context conditions (exercise in 1st vs. game in 1st), and the type of tests (reconstruction vs. oral recall). Vertical bars represent SEs. Ns for non-significant difference on *t*-tests comparing the type of contexts in each pair of conditions and * for significant difference at *p* < 0.05.

To summarize, only the interaction between the type of contexts and its order of presentation accounted for the results observed on the span scores. Children starting with the exercise condition (mean = 2.32, *SD* = 0.5) had a significantly lower span score during the game condition (mean = 2.04, *SD* = 0.5) presented afterward, *t*(50) = 3.04, *p* = 0.004. However, working memory performance in children starting with the game condition (mean = 2.27, *SD* = 0.5) did not differ in the exercise condition presented afterward (mean = 2.16, *SD* = 0.5), *t*(50) = 1.18, *p* = 0.25 (Figure 1).

## Discussion

In this study, our aim was to test the hypothesis that the joint effect of a highly meaningful context and a goal-oriented motor activity during a working memory task would influence children’s ability to maintain the goal and improve working memory performance. Moreover, this aid would be favorable to preschoolers only in the case of a reconstruction test, which favors goal neglect, whereas this aid should not affect an oral recall test that encourages goal maintenance in children. If such a combined help of a highly meaningful context with a goal-oriented motor activity in a reconstruction test can effectively boost goal maintenance, then we should observe a beneficial effect on preschoolers’ working memory performance. Our results did not support our hypothesis. First, type of contexts (game vs. exercise) and the type of tests (reconstruction vs. oral recall) did not affect memory performance, and no interaction was evidenced between these two variables. Only an interaction between the type of contexts and its order of presentation was significant. Children showed degraded working memory performance in the game condition when they started with an exercise condition. This detrimental effect was not observed when they started by the game condition followed by the exercise condition. The results thus appeared at odds with those of Istomina (1975) and Bertrand and Camos (2015), in which recall performance was improved in a game situation, which included both a goal cue and a motor activity, and this even with a reconstruction test. The results are also contradictory to Fitamen et al. (2019) who observed a benefit in 5-year-old children’s working memory performance in a situation combining a goal-oriented motor activity in a meaningful context. In the following, we examined the reasons why such a discrepancy appeared compared to the two previous sets of studies.

To understand the discrepancy in findings and the lack of beneficial effect in the reconstruction test, one can examine the design of the encoding phase. Brown (1975) has shown that children of 5 years of age are able to get similar memory performance in reconstruction and oral recall tests, when the temporal order of to-be-memorized information is in direct correspondence with the representation of its spatial order during the encoding phase. Concretely, this happens when children had to memorize items presented as pictures in a retention array in the same (spatial) order as they appeared (temporally) in a story told at the same time (Brown, 1975, Exp. 2). On the contrary, when the encoding does not make the link between spatial and temporal representations possible, which means that items occupied scrambled spatial positions when the story was presented, children obtained better memory performance in the reconstruction test compared to the oral recall test (Brown, 1975, Exp. 1). In the present study, a direct correspondence between the temporal and spatial orders can be built up during encoding. Indeed, by using a thin transparent bag at encoding, children had access simultaneously to the temporal representation of the order (one fruit or vegetable per second placed in the bag) and the spatial representation of this order (by looking at the column of fruits and vegetables in the bag). Based on Brown’s (1975) findings, our particular encoding condition can explain the absence of effect of the type of tests in the present study. This could also confirm that the link between temporal and spatial representations during encoding is critical in preschoolers, while this effect disappeared in older children (7–8 years of age) in study of Brown (1975, Exp. 1).

The present findings are also at odds with Fitamen et al. (2019) who reported a beneficial effect on working memory of the combination of contextual cues and action in 5-year-olds. Although the previous study and the present one shared the fact that the motor activity is related to the container of the items (the schoolbag in Fitamen et al., 2019; and the shopping stall in the present study), the main difference between the two studies is the implementation of the tasks. While we chose here a rather naturalistic setting akin to the daily activities of preschoolers (playing a shopping game, doing school-like exercise), Fitamen et al.’s (2019) task was computerized and presented on a tablet. Although further studies are required to examine in more details the divergence of findings, this discrepancy questions the transfer of effects observed in tablet to natural settings. In recent years, the use of tablets and computers to test young children became a norm and it provides several advantages for experimental psychology (e.g., better control of the conditions, collect of more fine-grained data). Nevertheless, the present study gave an example of how difficult it is to directly transfer knowledge from laboratory to classroom, and calls for more care when implications for practice are drawn from laboratory tests.

Finally, the absence of interaction effect between the type of contexts and the type of tests in the present study contradicts the idea that favoring goal maintenance has a decisive impact in 5-year-olds’ working memory performance, contrary to our hypothesis. Nonetheless, the interaction between the type of contexts and its order of presentation might indicate that performing a game situation first enabled children to effectively set the goal. Indeed, when the first condition is the game context, the context helps the goal identification thanks to the highly significant contextual characteristics of the game context (e.g., visual cues provided by the shopping stall, goal-oriented walk). The requirements of the memory task (e.g., remembering that the goal is to memorize, implementing maintenance strategies) can be transferred to the second (exercise) condition in which the goal was less salient. The grocery game condition, by giving a clearer meaning as to why memorizing shopping items (i.e., doing the shopping), could thus have served as a sort of tutorial that allow keeping the performance at the same level in the second (here exercise) condition. This tutorial effect could be beneficial thanks to contextualized learning. When performing the game condition first, children were engaged concretely in a meaningful activity. Then, they were able to transpose what they experienced in a living and concrete activity toward a more abstract activity, when doing the exercise as second condition. This enactment of the memorization situation is, moreover, one of the accounts suggested by Bertrand and Camos (2015) to explain the increase in working memory performance in a condition similar to the present game condition. On the contrary, working memory performance was reduced in the game condition when presented as second condition, for two reasons. First, children started with the exercise condition cannot benefit from the same kind of tutorial and contextualized learning as in the game condition, the goal of the task being less salient. Second, in the game condition, children had to process more information (e.g., understanding the story, looking at the shopping stall, and moving toward the stall), which could impair their memory capacity as their attentional resources need to be allocated to more information. This increased attentional demand added to the tiredness or weariness accumulated by the children during the first exercise condition may have been detrimental to working memory performance in the game condition when presented second. On the contrary, the high attentional demand induced by the game condition could have been adequately managed when this condition was presented first, and that attentional resources were still intact. In a follow-up study, the same type of context could be repeated within the same group of children (i.e., performing twice the game or exercise condition), to disentangle the effect of the condition from the potential effect of tiredness or weariness.

To conclude, the present study examined ways to improve working memory performance in preschoolers by providing contextual cues and motor activity. Contrary to laboratory testing condition, the implementation of the combination of contextual cues and motor activity did not benefit working memory performance in a more naturalistic setting. Nevertheless, the presentation at first of the task as a game seems to provide some information to preschoolers that they can transfer in a second attempt, contrary to the presentation as an exercise. Further studies are needed to strengthen this result and examine its determinants.

## Data Availability Statement

The datasets presented in this study can be found in online repositories. The names of the repository/repositories and accession number(s) can be found at: https://mfr.osf.io/render?url=https%3A%2F%2Fosf.io%2Fu89tm%2Fdownload.

## Ethics Statement

The studies involving human participants were reviewed and approved by the Ethics Committee of the University of Fribourg. Written informed consent to participate in this study was provided by the participants’ legal guardian/next of kin.

## Author Contributions

CF and VC wrote the manuscript. CF prepared the Figure 1. Both the authors contributed to the article and approved the submitted version.

### Conflict of Interest

The authors declare that the research was conducted in the absence of any commercial or financial relationships that could be construed as a potential conflict of interest.
